# Current Procedural Terminology Codes Combined With Hip and Femur Diagnosis‐Related Groups Provide a More Accurate Assessment of Costs

**DOI:** 10.1155/aort/5644038

**Published:** 2026-08-28

**Authors:** Robert A. Hymes, Daniel L. Rodkey, Max A. Schulman, Christopher Kuenze, Jeff E. Schulman, Jalen N. Broome, Sydney L. Payne, Greg E. Gaski

**Affiliations:** ^1^ Department of Orthopedic Surgery, Inova Fairfax Medical Campus, Falls Church, Virginia, USA, inova.org; ^2^ Madigan Army Medical Center, Tacoma, Washington, USA, mamc.amedd.army.mil; ^3^ Department of Kinesiology, University of Virginia, Charlottesville, Virginia, USA, virginia.edu; ^4^ Department of Orthopedic Surgery, Thomas Jefferson University, Philadelphia, Pennsylvania, USA, jefferson.edu

**Keywords:** CPT, Current Procedural Terminology, Diagnosis-Related Groups, DRG, femur, fracture, hip, total direct costs

## Abstract

**Introduction:**

Diagnosis‐Related Groups (DRGs) have become the basis of the Center of Medicare & Medicaid Services (CMS) hospital reimbursement system. Wide variability of patient factors and procedures bundled into a single DRG may contribute to significant cost variation and limited usefulness when analyzing economic performance. This investigation sought to analyze variation in hip and femur fracture inpatient costs as assessed by DRG and Current Procedural Terminology (CPT) codes.

**Materials and Methods:**

Patients age ≥ 18 treated with operatively managed fractures of the hip and femur at a Level 1 trauma center from 2019 to 2021 were retrospectively reviewed. Patients categorized as DRG 480, with major comorbidity/complication; 481, with comorbidity/complication; and 482, without comorbidity/complication were eligible. Seven common CPT codes were analyzed after exclusion of patients with low frequency CPT codes (< 50 instances). The primary outcome was total direct costs by DRG and CPT codes. Total direct costs were compared among CPT codes within each DRG using an ANOVA model. Costs were also compared among DRGs for each CPT code. Secondary outcomes include comparison of total direct costs by surgeon within DRG and within each CPT code.

**Results:**

A total of 896 patients were analyzed. Significant variation in costs was observed among different DRGs for specific CPTs (*p* < 0.001). Similarly, notable variation in total direct costs was recorded among different CPTs within each given DRG (*p* < 0.001). Although cost variations were observed between surgeon and DRG (*p* < 0.001), no significant interaction was observed once specific procedural (CPT) data were included in the model (CPT 27245: *F* = 1.077, *p* = 0.378; CPT 27506: *F* = 0.934, *p* = 0.504).

**Conclusions:**

While direct costs are influenced by the complexity of DRG assignment within the hip and femur fracture DRG family, the data may be limited in its applicability given the heterogeneity of patients and procedures captured within each DRG. Evaluating costs using a DRG–CPT combination, instead of DRG alone, may provide a more comprehensive and accurate understanding of costs of care.

**Level of Evidence:**

Economic and Decision Analyses Level III.

## 1. Introduction

Fractures of the hip and femur are common injuries with an incidence that has increased over the past 3 decades [1, 2]. This represents a significant economic burden for the US healthcare system as these patients are associated with high morbidity and mortality [3]. Trauma centers manage large numbers of young patients with high‐energy femur fractures and elderly patients with low‐ and high‐energy hip fractures [4, 5]. As concerns continue to grow around the financial viability of trauma centers, fiscal management of these patients is mandatory [6].

Hospital inpatients with fractures of the hip and femur who undergo surgical fixation are assigned into the same family of Diagnosis‐Related Groups (DRGs). The DRG system is a prospective payment system which categorizes patients with similar clinical characteristics and resource utilization. Instead of paying hospitals separately for specific services such as laboratory testing, diagnostic imaging, surgical procedures, medication, and hospital days, the payer (Center of Medicare & Medicaid Services [CMS] or private insurer) provides one bundled payment based on a patient’s assigned “DRG” category. The hospital receives a set payment regardless of the actual cost of care for a given patient. DRG is assigned based on primary diagnosis, demographics, procedures performed, patient comorbidities, and hospital complications. The DRG system incentivizes hospitals to provide efficient, value‐based care. The system has become the basis of the CMS hospital reimbursement system and has become a means of cost control [7]. Conversely, Current Procedural Terminology (CPT), established by the American Medical Association (AMA) in 1966, are codes used by physicians to report provided medical, surgical, and diagnostic services to health insurance companies [8]. Physicians are generally familiar with CPT, whereas hospital administrators predominantly communicate using DRG [9–12]. At present, there are 700 DRG codes and over 7000 CPT codes which precludes one‐to‐one mapping, rendering physician–administrator conversations regarding the finances of treating specific healthcare conditions confusing.

The usage of DRG and CPT has evolved beyond their original purpose; they are increasingly used for administrative tracking, pay for performance, guideline development, quality initiatives, bundled payments, hospital comparisons, and compensation models [8, 13, 14]. As disparate patients with a wide range of injuries are bundled into a single DRG, analysis by DRG alone less has become less useful [15]. Significant variability of patient factors and procedures within a single DRG makes it difficult to accurately assess physician economic performance solely by this system. Others agree and have called for the expansion of the DRG system [16, 17]. In contrast, the assessment of economic performance solely based on CPT is also insufficient as it does not consider patient complexity. The purpose of this study was to analyze total direct costs of inpatients at a Level I trauma center who underwent surgical repair of fractures of the hip and femur and to examine the interaction between the DRGs and CPTs.

The hypothesis was that there would be heterogeneity and significant cost variation among (1) different hip and femur CPTs within a given DRG and (2) different DRGs for a specific CPT. A secondary aim was to explore surgeon cost variation while considering DRG and CPT data.

## 2. Methods

Following Institutional Review Board approval (exemption with waiver of consent, U22‐02‐4676, May 1, 2025), a retrospective chart review was performed of patients aged ≥ 18 with an isolated hip or femur fracture at a Level 1 trauma center from January 2019 through December 2021. The institution is a high‐volume nonprofit tertiary care referral center with an academic environment providing training to two orthopedic surgery residency programs. The case‐mix index is 2.361. DRG categorization was recorded for each hip/femur fracture patient: 480, with major comorbidity or complication; 481, with comorbidity or complication; and 482, without comorbidity or complication [18]. DRG 480‐482 “excludes major joint” and thus hip fracture patients receiving arthroplasty treatment fall within DRG 521 (with major comorbidity or complication) or DRG 522 (without major comorbidity or complication). Examples of “comorbidity and complication” (DRG 481) include pneumonia, urinary tract infection, diabetes with complications, chronic kidney disease stages 3‐4, anemia, chronic obstructive pulmonary disease, electrolyte abnormalities, delirium, ileus, malnutrition, hypertension with complications, acute blood loss anemia requiring transfusion, and atrial fibrillation. Examples of major comorbidity and complication (DRG 480) include acute respiratory failure, septic shock, acute renal failure requiring dialysis, cardiac arrest, cardiogenic shock, coma, stroke, anoxic brain injury, coagulopathy, organ failure, and end‐stage renal disease with dialysis. A polytrauma patient was defined as any patient with a serious injury in two or more different anatomic regions, according to the Abbreviated Injury Scale. Specifically, those with additional operative fractures were excluded. Most polytraumatized patients did not meet inclusion criteria as they would have been assigned to DRGs within major trauma codes between 955 and 965. The manuscript was prepared in accordance with the Strengthening the Reporting of Observational Studies in Epidemiology (STROBE) reporting guidelines (Supporting information).

Patients with CPTs matching the inclusion criteria were identified from a physician practice billing database. All CPTs were originally entered by treating surgeons. DRGs were provided by hospital coding personnel and matched to the included patient encounters. Predetermined data points were extracted from the electronic health record by students. All clinical and financial data were verified by an orthopedic surgery resident (DR) and 3 surgeon authors (J.E.S., G.E.G., and R.A.H.).

Relevant resource and financial data were collected: length of stay (LOS), implant costs, and total hospital costs. Stryker Corporation (Kalamazoo, MI, USA) and DePuy Synthes, a Johnson and Johnson Company (Warsaw, IN, USA), implants were used by surgeons during the investigation period. Device costs were similar between companies. The highest frequency CPT codes of the cohort (minimum 50 occurrences) were matched for each patient to a hospitalization and associated DRG: 27235 percutaneous fixation femoral neck fracture, 27236 open reduction internal fixation (ORIF) femoral neck fracture, excluding arthroplasty, 27245 intramedullary nail (IMN) inter/subtrochanteric femur fracture, 27506 IMN femoral shaft fracture, 27507 ORIF femoral shaft fracture, and 27511/27513 ORIF extra/intra‐articular distal femur fracture, combined [19]. A minimum of 50 procedure events was used for concern that less frequently performed procedures may distract from the main intent of the investigation. Examples of excluded procedures were 27244 ORIF proximal femur with a plate (*n* = 14), 27470 nonunion femur repair without graft (*n* = 15), 27472 nonunion femur repair with graft (*n* = 25), and 27514 ORIF femur condyle (*n* = 8). Secondary CPT codes for hardware removal, debridement, and wound closure were excluded. Surgeries were performed by a six‐person, employed orthopedic trauma practice. Implant costs were derived from Horizon Performance Manager (HPM) cost accounting system. The charges represent specific implant costs that are stored in EPIC based on each type of implant. Total hospital costs are estimated using HPM, which allocates the patient care department cost from Oracle General Ledger to the charge codes generated in each patient care department.

### 2.1. Cost Comparison Between DRG and CPT

A chi‐squared analysis was used to describe the association between the frequency of CPT code usage and DRG assignment across all surgeons included in this analysis. Total direct costs were compared among CPT codes within each DRG using an ANOVA model. Total direct costs were also compared among DRGs for each CPT code. Main effects for both DRG and CPT code, as well as the interaction between DRG and CPT code, were reported. In the event of significant main effects, Tukey’s post hoc analyses were used to determine the effects of CPT code on total direct costs within each DRG. To support our primary comparative aim, we assessed homogeneity of variance using Levene’s test and normality using the Shapiro–Wilk test. We acknowledge that Levene’s test (*p* < 0.001) and Shapiro–Wilk test (*p* < 0.001) indicated violations of equal variance and normality assumptions, respectively. However, one‐way ANOVA is well documented as robust to these violations, particularly with adequate sample sizes, and given our research question centered on mean differences across groups, it remains the most appropriate analytic approach available.

A linear regression model was constructed to quantify how DRG and CPT codes jointly relate to total direct hospital costs. Total direct cost was used as the outcome and DRG group and CPT code were entered simultaneously as predictors. This approach allows us to estimate the independent contribution of each DRG and CPT category while holding the others constant. For DRG, the reference group is 482. For CPT, the reference group is 27235. The Shapiro–Wilk test indicated a violation of the normality assumption for model residuals (*W* = 0.717, *p* < 0.0001); however, linear regression is well documented as robust to nonnormality of residuals, particularly in large samples, and with *N* = 896, the central limit theorem ensures that inference on regression coefficients remains valid. Collinearity statistics were within acceptable limits for both predictors (VIF < 1.02), indicating no concern for multicollinearity.

### 2.2. Variation in Costs Between Surgeons

An exploratory analysis was conducted to better understand the effect of surgeon on total direct costs. Separate ANOVA models were used to investigate the effects of surgeon, CPT code, and DRG on total direct costs.

Finally, total direct costs between surgeons for the most prevalent CPT codes (27245 and 27506) in each DRG were compared using ANOVA models. Main effects for DRG and surgeon, as well as the interaction between DRG and surgeon, were reported. All statistical analyses were completed in Jamovi v2.2.5.0.

### 2.3. Sample Size Estimates

There were 2 sample size estimates run given our primary ANOVA (differences between CPT and DRG) and linear regression–based aims (association between CPT and DRG with costs). The omnibus ANOVA model reported the main effects and interactions for total direct costs among each combination of CPT and DRG (18 total groups). Assuming *α* < 0.05, and an acceptable statistical power (1−β) of 0.80 our final sample size (*n* = 896) would enable us to detect a small interaction effect (Cohen’s *d* = 0.30).

The linear regression model included 2 predictor variables (CPT code and DRG). Given 2 predictors, *α* < 0.05, and an acceptable statistical power (1−β) of 0.80, a minimum of 100 cases were required to detect small to moderate associations between predictors and the outcome variable (total costs).

## 3. Results

### 3.1. Patients

**FIGURE 1:**
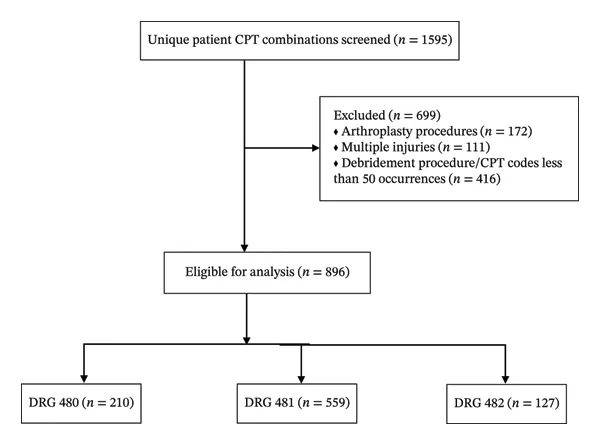
CONSORT diagram.

A total of 1595 unique patient–CPT combinations were screened, and 896 patients were eligible for final analysis. Patients were excluded for arthroplasty procedures (*n* = 172), multiple injuries (*n* = 111), and debridement procedures/CPT codes less frequent than 50 occurrences (*n* = 416) (Figure 1). There were 210 patients that fell in DRG 480, 559 in DRG 481, and 127 in DRG 482. The frequency of CPT codes within each DRG is detailed in Table 1. There was a significant difference in the frequency of CPT codes between DRGs (*X* ^2^ = 52.319, *p* < 0.001) (Table 1).

**TABLE 1:** Frequency of CPT codes within each DRG; *n* (%).

	DRG 480	DRG 481	DRG 482
CPT 27235 (*n* = 82)	14 (17.1%)	54 (65.9%)	14 (17.1%)
CPT 27236 (*n* = 54)	4 (7.4%)	29 (53.7%)	21 (38.9%)
CPT 27245 (*n* = 396)	98 (24.7%)	263 (66.4%)	35 (8.8%)
CPT 27506 (*n* = 193)	47 (24.4%)	107 (55.4%)	39 (20.2%)
CPT 27507 (*n* = 71)	16 (22.5%)	46 (64.8%)	9 (12.7%)
CPT 27511/3 (*n* = 100)	31 (31.0%)	60 (60.0%)	9 (9.0%)

### 3.2. Cost Comparison Between DRG and CPT

**FIGURE 2:**
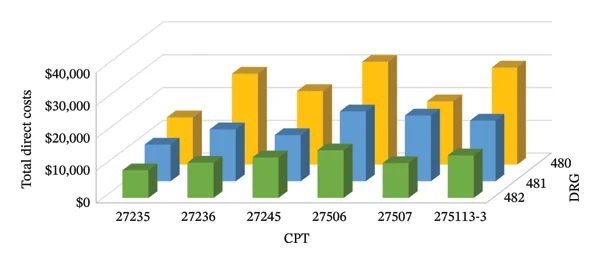
Graphical comparison of total direct cost analyzed by CPT code and DRG.

The combination of CPT code and DRG was significantly associated with total direct costs (*F* = 2.871, *p* = 0.002) (Table 2). When DRGs 480, 481, and 482 were combined, a significant association between total direct costs and CPT was observed (*F* = 17.053, *p* < 0.001) (Figure 2). Similarly, when all CPTs were combined, a significant association between total direct costs and DRG was noted (*F* = 43.064, *p* < 0.001) (Figure 2).

**TABLE 2:** Comparison of total direct costs by DRG and CPT code.

	DRG 480	DRG 481	DRG 482	*p* value
CPT 27235 (*n* = 82)	$14,498 ± 8213	$11,334 ± 2723	$8547 ± 1188	< 0.001
CPT 27236 (*n* = 54)	$27,829 ± 16,741	$15,994 ± 7161	$10,918 ± 1866	< 0.001
CPT 27245 (*n* = 396)	$22,531 ± 13,410	$14,258 ± 4750	$12,524 ± 3635	< 0.001
CPT 27506 (*n* = 193)	$31,460 ± 20,845	$21,480 ± 8678	$14,728 ± 3061	< 0.001
CPT 27507 (*n* = 71)	$19,389 ± 5773	$20,198 ± 5733	$10,781 ± 2190	< 0.001
CPT 27511/3 (*n* = 100)	$29,710 ± 16,720	$18,653 ± 5632	$13,145 ± 3296	< 0.001
*p* value =	< 0.001	< 0.001	< 0.001	

When examining specific CPT and DRG combinations, significant differences in mean total direct costs were observed among all CPT codes for DRG 480, 481, and 482; *p* < 0.001 (columns in Table 2). Significant differences in mean total direct costs were also observed among the 3 DRGs for several CPT codes; *p* < 0.001 (rows in Table 2).

Post hoc analysis revealed that DRG 480 ($24,915 ± 15,978) had higher total direct costs as compared to both DRG 481 ($16,406 ± 6738, *p* < 0.001) and DRG 482 ($12,417 ± 3465, *p* < 0.001) and DRG 481 had higher total direct costs when compared to DRG 482 (*p* < 0.001). With regards to cost analysis between CPT codes, 27235 (percutaneous fixation femoral neck) had lower direct costs ($11,398 ± 4356) compared to all other CPT codes (all *p* < 0.036).

A linear regression model demonstrated similar findings (Table 3). Total direct cost was used as the outcome and DRG group and CPT code were entered simultaneously as predictors. For DRG, the reference group is 482. For CPT, the reference group is 27235. Reported cost estimates represent additional cost compared with the reference procedures and patient complexity level. DRG 480 is associated with an increased cost of $12,746 (95% CI: $10,734–$14,758) compared to DRG 482. DRG 481 is associated with an increased cost of $4704 (95% CI: $2949–$6460) compared to DRG 482. CPT 27506 is associated with an increased cost of $10,704 (95% CI: $8399–$13,010) compared to CPT 27235. The model R2 is 0.26, indicating that 26% of variation in total direct costs may be explained by DRG and CPT codes alone.

**TABLE 3:** Linear regression model to quantify DRG and CPT relationship with total direct hospital costs.

	*β*	SE [95% CI]	*p*
DRG Code			
480–482	$12745.59	$1025.18 [10733.53, 14757.65]	< 0.001
481–482	$4704.19	$894.48 [2948.65, 6459.74]	< 0.001
CPT Code			
27236–27235	$5301.80	$1571.18 [2218.13, 8385.47]	0.001
27245–27235	$3749.16	$1082.64 [1624.34, 5873.99]	0.001
27506–27235	$10704.46	$1174.63 [8399.08, 13009.83]	< 0.001
27507–27235	$6778.10	$1443.20 [3945.61, 9610.59]	< 0.001
275113–27235	$8.687.26	$1330.16 [6076.62, 11297.89]	< 0.001

*Note:* Adjusted *R* ^2^ = 0.26.

### 3.3. Variation in Total Direct Costs Between Surgeons

The combination of DRG and surgeon was significantly associated with total direct costs (*F* = 2.610, *p* = 0.004). When examined independently, both surgeon (*F* = 5.335, *p* < 0.001) and DRG (*F* = 86.542, *p* < 0.001) were associated with cost variation (Table 4). This finding may be due to surgeon cost variability or could potentially be explained by procedural variability as CPT was not included here.

**TABLE 4:** Comparison of total direct costs between surgeons, within each DRG.

	DRG 480	DRG 481	DRG 482
Surgeon 1	$26,815 ± 13,928	$15,863 ± 5356	$10,933 ± 1585
Surgeon 2	$23,600 ± 12,274	$16,284 ± 5573	$12,424 ± 3285
Surgeon 3	$19,264 ± 7522	$15,905 ± 6993	$11,070 ± 2452
Surgeon 4	$28,578 ± 22,635	$17,307 ± 9342	$13,310 ± 3468
Surgeon 5	$20,153 ± 11,138	$15,662 ± 6207	$11,850 ± 3950
Surgeon 6	$33,016 ± 18,853	$17,354 ± 5961	$14,010 ± 4004

There was no significant interaction between CPT code and surgeon for total direct costs (*F* = 1.115, *p* = 0.318) (Table 5). This finding may be explained by minimal cost variance, and similar practice patterns, between surgeons in performing specific procedures. However, one may not assume that no surgeon cost variation exists as patient complexity (DRG) data were not included here.

**TABLE 5:** Comparison of total direct costs between surgeons, within each CPT code.

	CPT 27235	CPT 27236	CPT 27245	CPT 27506	CPT 27507	CPT 27511/3
Surgeon 1	$12,185 ± 3396	$20,979 ± 13,176	$15,002 ± 5521	$21,499 ± 12,041	$20,047 ± 7022	$21,570 ± 12,401
Surgeon 2	$10,170 ± 2621	$14,886 ± 3757	$17,730 ± 10,506	$19,835 ± 5701	$18.809 ± 3.391	$21,017 ± 9514
Surgeon 3	$10,507 ± 1910	$14,534 ± 12,317	$15,381 ± 5896	$21,795 ± 8121	$18,378 ± 1149	$20,041 ± 2682
Surgeon 4	$11,174 ± 2958	$11,776 ± 4185	$15,968 ± 8651	$26,503 ± 20,397	$17,372 ± 7034	$24,177 ± 15,742
Surgeon 5	$11,150 ± 3615	$11,705 ± 2426	$15,316 ± 9081	$19,958 ± 6487	$16,800 ± 5867	$17,647 ± 5719
Surgeon 6	$13,217 ± 8597	$17,659 ± 4973	$16,977 ± 9100	$22,452 ± 11,246	$23,450 ± 9482	$27,476 ± 14,278

To further explore the interaction between surgeon cost variation, DRG, and CPT, the two most common procedures were analyzed (27245 IMN hip and 27506 IMN femur). There was no significant interaction between DRG and surgeon within CPT code 27245 (*F* = 1.077, *p* = 0.378) nor CPT code 27506 (*F* = 0.934, *p* = 0.504) (Table 6). Although significant cost variability was observed between surgeon and DRG (Table 4, explained above), no significant interaction was observed once specific procedural CPT data were included in the model (Table 6). This suggests that surgeon cost variability observed in the DRG‐only analysis may be partially explained by an unequal distribution of specific procedures among surgeons as opposed to true surgeon practice variation.

**TABLE 6:** Comparison of total direct costs between surgeons and DRG within the two most common CPT codes.

CPT	Surgeon	DRG 480	DRG 481	DRG 482	*p* value
27245	Surgeon 1	$25,423 ± 14,462	$14,444 ± 5345	$12,303 ± 2940	*p* = 0.378
	Surgeon 2	$22,015 ± 8523	$14,027 ± 3832	$10,927 ± 949	
	Surgeon 3	$21,313 ± 13,439	$13,594 ± 3098	$9935 ± 1032	
	Surgeon 4	$18,176 ± 7764	$14,209 ± 3899	$11,526 ± 3737	
	Surgeon 5	$30,730 ± 20,652	$15,802 ± 5280	$12,740 ± 2535	
	Surgeon 6	$21,075 ± 14,532	$13,521 ± 5888	$13,725 ± 5178	

27506	Surgeon 1	$26,468 ± 7044	$19,620 ± 4712	$15,506 ± 2021	*p* = 0.504
	Surgeon 2	$32,561 ± 15,194	$18,680 ± 3831	$11,574 ± 1574	
	Surgeon 3	$38,916 ± 32,010	$25,488 ± 13,606	$15,244 ± 2648	
	Surgeon 4	$24,646 ± 5456	$22,516 ± 9537	$12,270 ± 623	
	Surgeon 5	$35,917 ± 25,075	$20,723 ± 3660	$17,798 ± 3500	
	Surgeon 6	$18,991 ± 6767	$20,393 ± 7281	$15,987 ± 2124	

## 4. Discussion

This review of 896 patients who underwent inpatient surgical procedures for hip and femur fractures found that the distribution of CPT codes varied significantly across different DRGs. For each CPT studied, there were significantly increased total direct costs among more complex DRG groups (480 > 481 > 482). This investigation also found significant differences in total direct costs among the seven most common hip and femur CPT codes within each DRG. These findings suggest that the choice of both the CPT code and the specific DRG significantly impact total direct costs, indicating a more nuanced and complex association between CPT codes and DRGs in the context of healthcare resource utilization and economic analysis. DRG captures patient complexity and is strongly associated with higher costs. CPT illustrates procedural variation and explains substantial additional cost differences within the same DRG. Using DRG alone oversimplifies cost attribution, while combining DRG and CPT provides a more accurate and clinically intuitive explanation of cost variation.

Understanding and navigating the complexities of healthcare finance are essential for hospital administrators and physicians. Based on their work, Samuel, et al. argued for reevaluation of the CMS DRG system, noting equal reimbursements between patients with complex pelvic trauma and patients with lesser pelvic and hip fractures [20]. Kelly et al. examined a small subset of patients with hip fractures and found that the CMS reimbursement for patients with intertrochanteric hip fractures was insufficient, disproportionately affecting healthier patients [21]. Both studies used diagnosis codes rather than DRGs as a basis for patient selection, limiting their findings. Similar to the current study population, Rose et al. detected a correlation between the range of surgical procedures within DRGs 480–482 and the resulting variation in hospital costs [12]. However, their analysis is limited in that they divided the entire cohort of patients into 3 categories based on surgical coding, thus recreating the arbitrary nature of the DRG system. A comparable study by Cairns et al. examined CMS claims from DRGs 480–482. Adjusting for age, comorbidities, and surgical procedure, they found an improvement in cost predictions compared to using DRGs alone [22]. Similarly, this study demonstrated that DRG analysis alone was insufficient for accurately measuring total direct costs secondary to the effect of the CPT codes. Baum et al. found that cost variation for inpatient spine surgery was largely surgeon driven and could not be clearly explained by the DRG classification system, leading to the development of a CPT‐based categorization which was more sensitive for predicting cost variations [11].

Similar to these reports, this study demonstrated significant differences in total direct costs across the different DRGs. DRG 480 is characterized as having the patients with major comorbidities and/or complications. In line with expectations, this group had higher average total direct costs compared to DRG 481, which represents patients with lesser comorbidities and/or complications. The healthiest patients were assigned to DRG 482 and had the lowest average total direct costs. Additionally, greater costs were observed for DRG 480 > 481 > 482 within each specific CPT studied (Figure 2 and Table 2). Patients with major comorbidities (DRG 480) cost, on average, approximately $11,000–$15,000 more than, otherwise, similar patients without comorbidities (DRG 482) (Table 3). Conversely, intramedullary nailing of a femoral shaft fracture (CPT 27505) costs approximately $9000–$13,000 more than percutaneous femoral neck fixation (CPT 27235), after accounting for DRG (Table 3).

Surgeon analyses revealed significant cost differences when only DRG data were considered (Table 4). This may be explained by true variation in practice patterns and implant utilization. Alternatively, this finding may be due to a difference in the number and type of procedures performed by one surgeon compared to another surgeon within a specific DRG as CPT information was not incorporated in this analysis. No statistically significant differences in cost variation between surgeons were observed in the CPT analyses (Table 5). Cost analyses based on CPT coding offer enhanced specificity not captured by DRG‐based analyses alone. Interestingly, analyses combining DRG and CPT coding for two of the most common procedures (IMN hip and IMN femur) revealed no difference in surgeon cost variation.

Despite observed cost differences among different DRGs, patient and procedural diversity may limit the usefulness of the data and hinder its applicability to surgeons making clinical decisions that drive the cost of care provided. Several factors contribute to the cost variability in trauma cases, including the severity of injuries, pre‐existing health conditions, and unforeseen complications during surgery. DRGs may capture patient complexity but encompass a large amount of procedural variation and should not solely be used to measure physician financial performance and cost efficiency. However, as CPTs were not designed to capture patient comorbidities, they also should not be used in isolation. The findings in this investigation provide valuable insight into the cost variations associated with different DRG and CPT codes and their combined influence on total direct costs. Incorporation of CPT financial data, in addition to DRG, may offer a more comprehensive understanding of cost variation, thereby leading to care decisions that could lead to opportunities for savings. Understanding this concept helps healthcare providers, administrators, and policymakers to communicate more clearly and assist in allocating resources effectively based on the needs and complexities associated with different patient groups.

The study population and associated DRG distribution are representative of a typical Level 1 trauma center. DRG family 480–482 was chosen because it represents the largest inpatient group of orthopedic trauma patients and best lends itself to the current data analysis. Studies that attempt to break down the complexities of the DRG system to practicing clinicians and raise awareness of the communication gaps between surgeons and hospital administrators are uncommon. The direct applicability of our conclusions is certainly a potential limitation of this study when considering all orthopedic inpatients across the country, particularly those in smaller community hospitals. DRG classification should not be considered the definitive classification of patients for cost analysis but rather a starting point. Failure to acknowledge this by hospital administrators will limit their ability to exact meaningful change at their institutions in the pursuit of value‐based care.

This investigation had several limitations. The retrospective nature of data collection prohibits identification of additional complicating factors that may have driven lengthy hospitalizations and cost of care. Analyses represented in Table 2 are subject to Type 1 error given the relatively low number of subjects for specific CPTs. The cohort was limited to patients with isolated injuries to reduce financial heterogeneity of polytrauma patients. Thus, the findings may not be extrapolated to multiply injured patients. Low‐frequency CPT codes were not available for statistical analysis and were thus excluded. Although there was minimal difference in implant costs between two implant vendors, an in‐depth implant analysis was not performed. CPTs are entered by surgeons and verified by coders. This practice is subject to variation and was not independently audited for this investigation. Similarly, DRGs are assigned by hospital coders and subject to variability. Although used throughout the health system, HPM cost estimations have not been validated from a clinical research standpoint. Finally, the findings from studying a single DRG family may not be representative of the entire DRG system.

The results of this study highlight the variability in frequency of CPT coding within a single DRG family. Significant cost differences were observed among different DRGs for specific CPTs. Similarly, notable variation in total direct costs was recorded among different CPTs within a given DRG. Although significant cost variation was observed when DRG and surgeon were analyzed, this difference evaporated when CPT was included in the model. The addition of CPT data offers enhanced specificity in cost analyses not captured solely by DRG. This study underscores the importance of considering these factors together for a more comprehensive understanding of the financial dynamics in surgical procedures. These results also contribute valuable information for understanding the cost implications of different procedures within the context of specific diagnoses. An improved understanding of cost variation may help administrators and providers direct resources where they are needed most, potentially leading to improved patient care. A future multicenter investigation may provide additional insight into the validity of the findings reported here. Additionally, future studies should consider incorporating additional patient‐ and injury‐specific factors such as age, comorbidities, injury severity, and complications.

## Funding

There were no funding sources for this investigation.

## Conflicts of Interest

The authors declare no conflicts of interest.

## Supporting Information

Additional supporting information can be found online in the Supporting Information section.

## Supporting information


**Supporting Information** Supporting Information: STROBE statement.

## Data Availability

Data used to support the findings of this study are available from the corresponding author upon reasonable request.
